# Orphan G Protein Coupled Receptors in Affective Disorders

**DOI:** 10.3390/genes11060694

**Published:** 2020-06-24

**Authors:** Lyndsay R. Watkins, Cesare Orlandi

**Affiliations:** Department of Pharmacology and Physiology, University of Rochester Medical Center, Rochester, NY 14642, USA; Lyndsay_Watkins@URMC.Rochester.edu

**Keywords:** G protein coupled receptor (GPCR), G proteins, orphan GPCR (oGPCR), mood disorders, major depressive disorder (MDD), bipolar disorder (BPD), anxiety disorders, antidepressant, animal models

## Abstract

G protein coupled receptors (GPCRs) are the main mediators of signal transduction in the central nervous system. Therefore, it is not surprising that many GPCRs have long been investigated for their role in the development of anxiety and mood disorders, as well as in the mechanism of action of antidepressant therapies. Importantly, the endogenous ligands for a large group of GPCRs have not yet been identified and are therefore known as orphan GPCRs (oGPCRs). Nonetheless, growing evidence from animal studies, together with genome wide association studies (GWAS) and post-mortem transcriptomic analysis in patients, pointed at many oGPCRs as potential pharmacological targets. Among these discoveries, we summarize in this review how emotional behaviors are modulated by the following oGPCRs: ADGRB2 (BAI2), ADGRG1 (GPR56), GPR3, GPR26, GPR37, GPR50, GPR52, GPR61, GPR62, GPR88, GPR135, GPR158, and GPRC5B.

## 1. Introduction

Mood alterations due to pharmacological treatments that modulate serotonergic and noradrenergic systems laid the foundations for the monoamine hypothesis that has led research on mood disorders since the late 1950s [1,2,3]. Dopaminergic alterations have also been associated with major depressive disorder (MDD) symptoms, such as anhedonia [4]. Later, the involvement of the hypothalamic–pituitary–adrenal (HPA) axis was identified, providing a connection between environmental and psychological stressors and the development of affective disorders [5]. Morphological studies in animal models, together with neuroimaging in MDD patients, demonstrated that stress-induced depression is associated with the atrophy of important limbic and cortical brain regions, characterized by the reduction in dendritic arborization, the number of spines, and functional responses [6,7,8,9,10,11,12]; however, increased and sustained amygdala activity, the brain region responsible for the processing of emotions, was also demonstrated, suggesting an altered connectivity among all of these brain areas [7,13,14,15]. Many of these regions express high levels of glucocorticoid receptors and are therefore regulated by the HPA axis through the action of stress hormones on transcriptional programs [16,17]. More recently, evidence of the involvement of further signaling pathways and receptor systems prompted the drug discovery enterprise to move beyond the monoaminergic systems. Particularly, the observation that the glutamatergic modulator, ketamine, elicits fast-acting antidepressant responses has driven an intensive research effort that culminated with the recent approval of ketamine for treatment-resistant depression, not without raising concerns about its safety [18,19,20,21,22]. The pharmacological treatment of patients diagnosed with bipolar disorder (BPD) consists of mood stabilizing medicines, with lithium remaining the most effective treatment almost seventy years after its serendipitous discovery [23]. The most accredited hypothesis on the mechanism of action of lithium treatments involve the inhibition of inositol monophosphatase and glycogen synthase kinase 3, together with the regulation of calcium signaling, neuroplasticity, neurogenesis, G protein-activated potassium channels, as well as an association with long non-coding RNAs [24,25,26,27,28]. Finally, mood disorders often show comorbidity with anxiety, which is sometimes treated pharmacologically with antidepressants as an alternative to anxiolytic drugs, which indicates the presence of common biological substrates [29]. However, the first choice of pharmacological treatment for anxiety disorders consists in benzodiazepines, in spite of the risk of dependence if taken over a long period of time. The mechanism of action of benzodiazepines involves the hyperpolarization of neurons, which is achieved by potentiating the action of γ-aminobutyric acid (GABA) on the ligand-gated chloride channels GABA_A_ [30]. With GABA as the main inhibitory neurotransmitter in the central nervous system, benzodiazepine action decreases the firing of action potentials counteracting the neuronal hyperexcitability classically associated with anxiety [30]. In conclusion, despite strong evidence supporting models that include a dysregulation of monoaminergic, glutamatergic, and HPA systems, which are not mutually exclusive, a compelling and unified description of the neurobiology of affective disorders and the mechanisms of action of the medicines used to treat them are still unavailable. 

G protein coupled receptors (GPCRs) are 7-transmembrane, metabotropic receptors involved in the regulation of nearly every physiological process. The large GPCR family constitutes the most exploited drug target in the human genome [31,32]. For instance, GPCRs mediate the signaling of neurotransmitters that are targeted by the action of many antidepressants [33,34]. Specifically, treatments with selective serotonin reuptake inhibitors (SSRI) and norepinephrine reuptake inhibitors (NRI), the most common classes of antidepressants, result in an indirect stimulation of serotonin (5-HT) and norepinephrine (NE) GPCRs. Naturally, several clinical and preclinical studies pointed at the modulation of a variety of 5-HT receptors among the mechanisms of action of SSRIs [35]. Similarly, several α-adrenergic receptors for NE have been implicated in the pathophysiology of MDD [36]. However, the pharmacological targeting of single receptors failed to prove an efficient therapy in clinical trials. Over the years, many more GPCRs have been explored as pharmacological targets for the action of novel antidepressants or have been associated with the pathophysiology of MDD and anxiety [33,37,38,39]. Orphan GPCRs (oGPCRs) constitute a subfamily of GPCRs whose endogenous ligands have not yet been identified. oGPCRs represent an important portion of the so-called dark druggable genome, a large group of understudied genes for which biochemical tools and assays are underdeveloped [40]. According to the recommendations of the International Union of Basic and Clinical Pharmacology Committee on Receptor Nomenclature and Drug Classification (NC-IUPHAR), an oGPCR is considered de-orphanized when reproducibility and in vivo pairing likelihood criteria are met [41,42]. This review focuses on oGPCRs that currently do not meet the NC-IUPHAR criteria and are therefore active subjects of research.

The generation of animal models overexpressing or lacking the expression of specific oGPCRs is a widely used approach to explore their role in mood disorders. Despite the limits in interpreting altered mice behavior as a translation of human affective disorders, numerous tests have been validated pharmacologically and represent a powerful tool to investigate the core symptoms of depression. Most of the tests for depressive-like behaviors in rodents are based on either learned helplessness, the development of a passive behavior, or behavioral despair, measured as failure to continue in escape-directed efforts after stress. Both the tail suspension test (TST) [43] and the forced swim test (FST) [44] use the measurement of immobility time as an index of a rodent’s effort to escape [45], while the novelty-suppressed feeding test (NSFT) [46,47] relies on the innate fear of rodents for novelty itself. Anhedonia represents a different aspect of depression that is generally measured in rodents using the sucrose preference test (SPT) [45,48]. On the other side, some of the most widely applied tests for anxiety-related behaviors in rodents are the elevated plus maze (EPM) [49] or elevated zero maze (EZM) [50], the light-dark transition test (LDT) [51], the marble burying test (MBT) [52], and the open field exploration test (OFT) [53], which are all based on the rodents’ innate aversions to brightly illuminated open areas. These tests have been validated by treatments with anxiolytic drugs. The advantages and drawbacks of each test have been reviewed elsewhere [54,55]. Overall, the behavioral analysis of animal models by applying a combination of the tests remains a fundamental resource to achieve new knowledge on the roles of oGPCRs and their potential to help understand the molecular basis of mood disorders in humans. Unbiased -omics approaches have also been attempted to explore the overall effect of antidepressant treatments, as well as the effect of chronic stress paradigms in animal models [56,57,58]. In addition, comprehensive transcriptomics analyses of post-mortem brain tissues from patients have been carried out to evaluate global changes in gene expression levels [59,60,61]. Finally, genome wide association studies (GWAS) have been addressing the genetic architecture of mood disorders [62,63,64]. This systems biology approach helped revealing the involvement of many oGPCRs in the neurobiology of affective disorders, therefore uncovering alternative potential therapeutic targets.

## 2. Systematic Analysis of oGPCRs in Anxiety and Mood Disorders

In the following section, we summarize the experimental evidence linking oGPCRs to symptoms of anxiety, mood disorders, or their pharmacological treatment, as shown in Table 1. Among these oGPCRs, nine belong to the GPCR class A (G protein coupled receptor 3 (GPR3), GPR26, GPR37, GPR50, GPR52, GPR61, GPR62, GPR88, and GPR135), two to the class C (GPR158 and GPRC5B), and two are adhesion GPCRs (ADGRB2/BAI2 and ADGRG1/GPR56). We discuss proposed mechanisms explaining the role of each oGPCR in modulating cellular and molecular processes within brain circuits, whose deficits promote stress-related disorders and anxiety. Finally, we collect information about expression patterns in the brain, cell-specificity, the modulation of signaling pathways, regulation by stress hormones, and changes in response to antidepressant treatments in humans and animal models. 

### 2.1. ADGRB2 (BAI2)

The Brain-specific Angiogenesis Inhibitor 2 (BAI2 or ADGRB2) was the first adhesion GPCR to be associated with mood disorders in animal studies [65]. ADGRB2 expression is predominantly found in the brain, specifically in neurons and astrocytes of the hippocampus, amygdala, and cerebral cortex [96,97]. The mechanism of activation of ADGRB2, which appears to be common with other members of the adhesion GPCR family, includes the self-cleavage of its long extracellular domain [98,99,100]. After cleavage, a tethered ligand is exposed and can change the conformation of the receptor to a G protein activating state [98]. In vitro, once the extracellular region is cleaved at the GPCR proteolytic site, truncated ADGRB2 specifically activates the Nuclear Factor of Activated T-cells (NFAT) luciferase reporter, suggesting a coupling to Gαq [101]. However, following signaling and biochemical studies of the activating mutation R1465W, it was revealed that ADGRB2 stimulates the NFAT pathway by Gβγ liberation and the activation of calcium channels and not through Gq [102]. Further in vitro analysis using the G protein inhibitors, pertussis toxin (PTX) and gallein, pointed instead to a coupling to Gαi/o/z family members, with a preference for Gαz [102].

**Figure 1:**
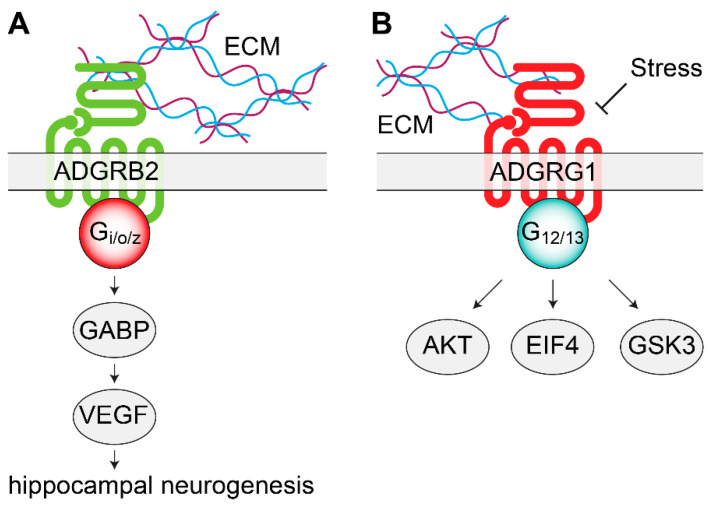
Signaling mechanisms of adhesion orphan G protein coupled receptors (oGPCRs) involved in mood disorders. (**A**) ADGRB2 interacts with the ECM to activate Gi/o/z proteins. In the dentate gyrus of the hippocampus, ADGRB2 activates intracellular GABP and VEGF, leading to increased neurogenesis. (**B**) Stress decreases ADGRG1 mRNA levels. By coupling with G12/13, ADGRG1 modulates AKT, GSK3, and EIF4 signaling pathways, intracellular signaling molecules previously associated with depression and with the action of antidepressants. AKT, protein kinase B; ECM, extracellular matrix; EIF4, eukaryotic initiation factor 4F; GABP, GA-binding protein; GSK3, glycogen synthase kinase 3; VEGF, vascular endothelial growth factor.

After creating a knock out (KO) mouse model, it was found that deficiency in ADGRB2 induced an antidepressant-like state [65]. The motor activity of the ADGRB2 KO mice during a social defeat stress paradigm was significantly higher compared to wild-type littermates, indicating a resilience to stress-induced depression [65]. ADGRB2 KO immobility time during the TST was also significantly lower compared to the wild-type controls, suggesting antidepressant-like behavior [65]. A battery of behavioral tests showed that the ADGRB2-deficient mice displayed normal motor activity, spatial learning, and memory, and did not show anxiety-related behaviors in the OFT [65]. Okajima and colleagues also identified a greater level of neurogenesis in the dentate gyrus of the hippocampus in ADGRB2 KO mice versus wild-type controls [65]. According to the hypothesis that a reduction in hippocampal neurogenesis contributes toward depression, while increasing neurogenesis, could be part of the mechanism of action of antidepressant treatments [103], it was speculated that the antidepressant-like behavior observed in the ADGRB2 KO mice could depend on this process. It was also discovered that ADGRB2 regulates the transcription of vascular endothelial growth factor (VEGF) by activating the repressor GA-binding protein [104]; as such, ADGRB2 helps regulate angiostatic function in the brain, resulting in an inverse correlation between the expression levels of ADGRB2 and VEGF [96]. These findings pointed to the ADGRB2 regulation of VEGF as a potential pathway to control hippocampal neurogenesis and, therefore, alter mood-related behaviors [22,65]. Such an intriguing hypothesis remains to be tested directly, as shown in Figure 1.

### 2.2. ADGRG1 (GPR56)

Recent findings have shown the association of another adhesion GPCR with depression—ADGRG1 (GPR56) [66]. As described for ADGRB2, the mechanism of ADGRG1 activation involves the self-cleavage of its long ectodomain to expose a stimulating tethered ligand [98,99]. Using in vitro luciferase reporter assays, several groups explored ADGRG1 signaling properties and showed a primary coupling to Gα12/13, which initiates downstream RhoA activation [98,105,106,107]. Extracellular interaction with collagen III seems to be part of the activation mechanism of ADGRG1 [108] and should be considered when investigating the physiological roles of this adhesion GPCR. Across the brain, ADGRG1 is expressed both in neurons and glia, and it regulates many processes that may be related to antidepressant responses [109,110,111,112,113], as shown in Figure 1.

ADGRG1 mRNA in blood cells was up-regulated in response to a variety of antidepressant treatments, but, importantly, only in patients responding to the therapy and not in respondent patients treated with placebo [66]. Remarkably, transcriptomic studies on post-mortem brain tissues from MDD patients and control subjects revealed a significant down-regulation of ADGRG1 in the dorsolateral PFC [59]. Supporting these results, animal studies demonstrated that unpredictable chronic mild stress (UCMS) down-regulated ADGRG1 mRNA in blood cells, as well as in PFC [66]. Treatments with the SSRI fluoxetine were able to rescue this stress-induced phenotype in mice, at the same time leading to an up-regulation in ADGRG1 [66]. As direct evidence that ADGRG1 was not only modulated by antidepressant treatments, but was mediating part of the therapeutic effects of antidepressant drugs, the viral over-expression of ADGRG1 in the prefrontal cortex (PFC) induced antidepressant-like effects in mice tested with TST and FST. On the contrary, the viral knockdown of ADGRG1 in mouse PFC induced depressive-like symptoms in TST and FST, but also anhedonia, measured using the SPT. Interestingly, ADGRG1 knockdown induced an anxiety-like phenotype in the EZM [66]. Fluoxetine treatments were shown to reduce the immobility during the TST in wild-type mice, while they were not effective in ADGRG1 knockdown, again suggesting a direct role of ADGRG1 in the mechanism of action of this antidepressant [66]. As further evidence, the exogenous activation of ADGRG1 by the infusion of activating peptides [114] in mouse PFC generated antidepressant-like effects that were not obtained by infusion in the nucleus accumbens, indicating a specific role of ADGRG1 expressed in the PFC [66]. Finally, ADGRG1 agonist peptides, applied to neuroblastoma cell cultures, up-regulated the AKT/GSK3/EIF4 pathways [66]. Such pathways are modulated by stress, and are also known targets of the action of commercial antidepressants [115,116,117,118]. Overall, ADGRG1 was concluded to be a potential antidepressant target since it is involved in various biological functions relevant to the pathophysiology of depression: neurogenesis, oligodendrocyte development, progenitor cell migration in brain, and myelin repair [109,110,119]. At the same time, the observation that ADGRG1 is up-regulated in the blood cells of MDD patients that respond to antidepressants suggests its quantification as a peripheral biomarker of the efficacy of treatments.

### 2.3. GPR3

First cloned in 1994 [120,121], GPR3 is expressed in the mouse brain structures involved in stress-related behaviors: habenula—where it shows the highest expression—hippocampus, amygdala, limbic system, and cortex [67]. When overexpressed in COS-7 and CHO cell lines, GPR3 stimulated the endogenous adenylate cyclase in the absence of any agonist, which suggested coupling to Gαs, and high levels of constitutive activity, which suggested the original name of adenylate cyclase constitutive activator (ACCA) [122]. Further studies in rodent cerebellar granule neurons (CGNs), rodent oocytes, and HEK293 cells confirmed the Gαs-coupling of GPR3 [123,124,125,126]. In 2002, sphingosine 1-phosphate was proposed as an endogenous ligand for GPR3 [123,127]; however, these results were not reproduced and, accordingly, this ligand-oGPCR pairing is still debated [67,128].

Since GPR3 has been shown to modulate cAMP levels in brain regions which can contribute to stress-related behaviors [129], a line of GPR3 KO mice was developed to study its potential role in mood disorders [67]. OFT was conducted on adult male GPR3 KO and wild-type mice in order to gauge their exploratory behavior and uncover an anxiety-like behavior in GPR3 KO mice [67]. Additionally, during EPM tests, the GPR3 deficient mice showed less time spent in the open arms and a lower percentage of entries, which are also associated with an anxiety-like behavior that could be alleviated by treatment with the anxiolytic diazepam [67]. An analysis of despair behaviors using FST and TST revealed a longer duration of immobility in the GPR3 deficient mice, compared with the wild-type littermates; such a depressive-like phenotype was rescued by treatment with fluoxetine [67]. Finally, the GPR3 KO mice displayed no differences in learning capability nor adaptiveness when compared to the wild-type controls [67]. In an effort to explain the molecular mechanisms leading to the behavioral deficits of the GPR3 KO mice, Valverde and colleagues evaluated the function of the HPA axis by measuring the serum corticosterone levels in basal conditions and after the acute stress produced by TST. The results showed comparable basal corticosterone levels. Furthermore, after the mice were acutely stressed, the corticosterone levels increased similarly in the wild-type and GPR3 deficient mice [67]. Since the HPA axis was not involved in the observed anxiety- and depressive-like behaviors in the GPR3 KO mice, Valverde and colleagues next conducted a neurochemistry analysis specifically looking at the hypothalamus, frontal cortex, and hippocampus. The GPR3 KO mice showed a deficit in 5-HT, NE, and dopamine (DA) levels within multiple brain regions. The hypothalamus and frontal cortex contained lower levels of 5-HT and NE with similar amounts of DA, whereas 5-HT and DA levels were lower in the hippocampus, without significant differences in NE levels [67]. It was recently shown in rodent CGNs that GPR3 expression activates the AKT, ERK, and PKA signaling pathways [130,131]. While these results were obtained in rodent CGNs, the same signaling cascades are known to contribute towards mood disorders in other brain areas [129,132,133]. In this direction, the primary culture of hippocampal neurons showed lower basal intracellular cAMP levels in the GPR3 KO mice compared to the wild-type littermates [67]. GPR3 is among the several GPCRs modulated by cannabidiol (CBD), which acts on GPR3 as an inverse agonist [134]. Within the last ten years, research on CBD has revealed that both acute and chronic CBD administration elicits antidepressant and anxiolytic effects in rodents [135]. Research aimed at testing the hypothesis that GPR3 mediates at least part of the antidepressant effects attributed to CBD has not yet been performed. Overall, looking into the GPR3 modulated signaling pathways in cell types within brain regions that are relevant for mood-related behaviors could provide new insights into the neurophysiological role of GPR3.

### 2.4. GPR26

GPR26 is a highly conserved oGPCR, first cloned in 2000 [136]. Because of GPR26’s sequence homology with purinergic P2Y receptors, it was hypothesized to be possibly activated by nucleoside di- and tri-phosphates [136]; however, the results of such studies were negative, leaving GPR26 an orphan [136]. It was also found that GPR26 overexpression in HEK293 and Rh7777 cells induced a significant increase in basal cAMP levels, suggesting high levels of constitutive acitivity and Gs coupling [136]. This data were later validated in HEK293 cells where transfections of GPR26, along with a cAMP response element (CRE) luciferase reporter, were conducted in a dose-dependent manner, showing a direct correlation between luciferase activity and the amount of transfected GPR26 plasmid [137]. Moreover, GPR26 coupling to Gs was further confirmed with a chimeric yeast system [137]. The expression of GPR26 is brain specific: in rats, the areas of highest expression are the striatum, pons, cerebellum, cortex, hippocampus, and hypothalamus [68,136]; similarly, in mice, GPR26 is expressed in the whole brain but enriched in the amygdala, hippocampus, and cortex [68,137]; in the human brain, GPR26 is expressed in the amygdala, hippocampus, and thalamus [137].

In 2011, Zhang and colleagues created and characterized a GPR26 KO mouse strain. While these mice displayed no gross abnormalities compared to the wild-type littermates, they did show anxiety- and depressive-like behaviors, which was concluded after a series of behavioral experiments [68]. OFT revealed that GPR26 KO mice spent less time in the center of the arena compared to the wild-type littermates, a raw measurement of anxiety-like behaviors in rodents. EPM tests, another method for evaluating rodent anxiety, yielded similar results with the GPR26 KO mice, displaying reduced open-arm exploration, measured as distance and time, compared to the wild-type littermates [68]. FST and TST tests were implemented to evaluate depression-like behaviors in mice. In the TST, the GPR26 KO mice displayed a statistically longer immobility time compared to the wild-type littermates, while the FST showed a similar trend without reaching statistical significance (*p* = 0.051) [68]. In addition, the GPR26 KO mice showed no significant difference in spatial learning or memory abilities when compared to the wild-type mice, suggesting a specific role of GPR26 in mood regulation [68]. However, the GPR26 KO mice exhibited a higher preference for ethanol in a two-bottle free-choice paradigm, which indicates further neurophysiological roles for this oGPCR [68]. Not surprisingly, stress disorders and anxiety often show comorbidity with alcohol drinking behaviors [138,139]. Since GPR26 has been shown to affect cAMP levels [136,137], Zhang and colleagues investigated the phosphorylation of the cAMP response element binding protein (CREB), which is regulated by the cAMP-PKA pathway, in the GPR26 KO mouse by Western blot in the whole brain, cortex, hippocampus, and cerebellum, and by immunohistochemistry in the amygdala. Total CREB levels were comparable between genotypes; however, the levels of phospho-CREB in the central amygdala (CeA) were significantly lower [68]. Due to GPR26 being brain-specific with high expression levels in brain areas affecting mood regulation, studying this oGPCR and the signaling pathways that it modulates may provide useful insights for the treatment of anxiety and depression.

### 2.5. GPR37

GPR37 is a glia-enriched oGPCR implicated in many neuropathologies, such as MDD, BPD, autism, and Parkinson’s disease [59,140,141]. Despite indications of GPR37 pairing with four peptides—head activator (HA) [142], prosaposin (PSAP) [143], bioactive lipid neuroprotectin D1 (NPD1) [144], and regenerating islet-derived family member 4 (Reg4) [145]—certain and reproducible proofs of receptor activation are still missing [146]. Recently, it has been proposed that a native cellular environment is a requirement to obtain proper GPR37 activation [147]. Nonetheless, several reports have shown that the PSAP, HA, and NPD1 stimulation of cells transfected with GPR37 induced PTX-sensitive effects, indicating Gi/o coupling [142,143,144]. In the human brain, GPR37 is enriched in the corpus callosum, substantia nigra, caudate nucleus, hippocampus, and medulla oblongata [148].

The first evidence of an involvement of GPR37 in mood disorders resulted from a microarray gene expression analysis of the temporal cortex obtained post-mortem from MDD patients that demonstrated a lower expression of GPR37 [69]. An analogous transcriptional profiling of post-mortem dorsolateral PFC and the anterior cingulate cortex (ACC) from MDD and BPD patients using gene microarray showed significant changes in GPR37 expression with a lower expression in the ACC of MDD patients, and a higher expression in the ACC and PFC of BPD patients [59]. These results were validated afterwards by quantitative real time PCR (qRT-PCR) in the ACC [59]. A further qualitative characterization by in situ hybridization showed that GPR37 mRNA expression was more prominent in the ACC sections from patients with a diagnosis of BPD, whereas it was lower in the ACC sections from MDD subjects, compared to controls [59]. Further studies found that GPR37 gene expression was also downregulated in the amygdala of MDD subjects [71]. Such results were replicated in a rodent model of depression, where mice underwent a UCMS protocol and were treated with two classes of antidepressants. Gene array expression studies in several brain regions revealed a downregulation of GPR37 in the amygdala that was reversed by treatments of fluoxetine or the antagonist of corticotropin-releasing factor-1 (CRF1) receptor, SSR125543 [70]. Furthermore, two independent animal studies using GPR37 KO mice revealed a role for GPR37 in anxiety- and depression-like phenotypes, although controversial results emerged [72,73]. The first study, by Mandillo and colleagues, analyzed male and female GPR37 KO mice compared to their wild-type littermates in a battery of tests for anxiety-like and depressive-like behaviors, and they investigated if a particular phenotype could be influenced by age [72]. From their study, it emerged that aged female GPR37 KO mice showed a significantly lower number of entries and time spent in the open arms of an EPM, compared to the wild-type littermates [72]. However, adult females and aged males did not show significant differences in the EPM, suggesting the age and sex specific effects of GPR37 deficiency on anxiety-like behaviors [72]. Moreover, the adult and aged female mice did not show statistically significant differences between genotypes in the OFT and LDT, limiting the role of GPR37 to aged females, and in the EPM only [72]. The further observation that only aged female GPR37 KO mice showed a significant higher immobility in the FST supports the sex and age specific roles of GPR37 [72]. A neurochemistry analysis of the monoamine and amino acid neurotransmitter levels in aged mice of both sexes revealed reduced levels of 5-HT, DA, and GABA in the olfactory bulb, as well as an increase in DA levels in the striatum, only in the GPR37 KO female mice [72]. The authors concluded that the lack of GPR37 induced anxiety- and depressive-like phenotypes in the aged females only [72]. In a second study, Lopes and colleagues utilized MBT and EPM to investigate anxiety-like behaviors and the signaling consequences of knocking-out GPR37 in mice [73]. In their report, male GPR37 KO mice displayed an anxiolytic behavior with less marbles buried in the MBT and a significantly greater time spent in the open arms in the EPM [73]. Interestingly, the anxiolytic behavior observed was completely reversed by systemic treatment with the adenosine A_2A_ receptor (A_2A_R) antagonist, SCH 58261. The authors suggested, therefore, a functional interaction between A_2A_R signaling and GPR37 within the hippocampus [73]. Finally, using an innovative optogenetic approach, Zheng and colleagues revealed new aspects of GPR37 signaling related to anxiety-like behaviors. By replacing the intracellular portions of channelrhodopsin2 (ChR2) with the corresponding amino acid sequences of GPR37, they designed a ChR2-GPR37 chimera, aimed at bypassing the lack of a ligand to activate GPR37. First, they validated the approach by measuring the intracellular decrease in cAMP levels and the augment in ERK phosphorylation in the light-activated transfected HEK293T cells [74]. Then, the viral delivery of the ChR2-GPR37 chimera in the dorsomedial striatum allowed them to trigger GPR37-driven signaling cascades that resulted in an increased time spent in the center during the OFT, indicating an anxiolytic behavioral response [74]. Overall, overwhelming evidence from both human and animal studies indicate a modulation of GPR37 expression levels by events that trigger affective disorders, while its role in mediating behavioral consequences is still debated and requires further research.

### 2.6. GPR50, GPR61, GPR62, and GPR135: Orphan Receptors That Modulate the Melatonergic System

Melatonin, a hormone produced by the pineal gland at night, has been extensively studied for its role in the modulation of the sleep–wake cycle and circadian rhythm in humans [149]. Since depression has been linked to disturbances in sleep [150], melatonin and the two GPCRs it activates, MT1 and MT2, have been studied as targets for mood disorders [75,151,152,153]. While melatonin production is restricted to a single area, MT1 and MT2 receptors are expressed in a multitude of locations: brain, retina, cardiovascular system, liver, kidney, platelets, and ovary/granulosa cells, to name a few [154,155]. As with many other GPCRs, melatonin receptors have been shown to homomerize, but are also capable of forming heteromers between them and with a small class of oGPCRs in a way that increases the variety of signaling pathways that they can initiate [156]. This group of oGPCRs—GPR50, GPR61, GPR62, and GPR135—show specific expression patterns in the brain that are partially overlapping with those of melatonin receptors MT1 and MT2; however, cell-specific co-expression studies in the brain have not been performed yet. Remarkably, none of these oGPCRs seem to be directly activated by melatonin [157]; instead, they have been shown to modulate melatonin receptor signaling by heteromer formation [156]. GPR50 (also known as H9 or ML1X) shares the highest sequence homology with melatonin receptors [158], and it is an X-linked oGPCR with high expression in the hypothalamus, pituitary, and adrenal glands in humans, rodents, and sheep [159,160,161,162]. GPR50 forms heteromer complexes with both MT1 and MT2; however, while MT2 function is not affected by heteromerization with GPR50, the engagement of GPR50 with the MT1 receptor inhibits the melatonin from binding, at the same time blocking any downstream signaling cascades [163]. According to this study, the inhibition was due to the long intracellular C-tail of GPR50, which most likely blocked the recruitment of β-arrestin and the coupling to Gq and Gi/o proteins [163]. GPR61 expression was found in the human brain—caudate, putamen and thalamus—while a more widespread expression was reported in rat brains—cortex, hippocampus, thalamus, hypothalamus, and midbrain [164]. The expression of GPR62 was found in the basal forebrain, frontal cortex, caudate, putamen, thalamus, and hippocampus [164], and while GPR135 can be found in various tissues (i.e., eyes and peripheral tissue), this oGPCR is also expressed in the pituitary gland [165,166]. Although these oGPCRs do not directly interact with melatonin, they do heteromerize with MT2 and regulate part of its function [157]. Similar to the GPR50/MT1 heteromers, the MT2 complexes with the other three oGPCRs inhibit β-arrestin recruitment; however, unlike GPR50/MT1, MT2 heteromers continue to allow Gi/o signaling to occur [157]. Additionally, it was found that GPR61 and GPR62 displayed constitutive Gs signaling activity, resulting in higher levels of cAMP [157,167]. When these receptors were co-expressed with MT2 in the HEK293 cells, the higher cAMP levels in cells expressing GPR61 or GPR62 were dose-dependently lowered by the presence of melatonin. The relationship between GPR61 or GPR62 with MT2 suggests a level of cross-talking between the receptors [157].

**Figure 2:**
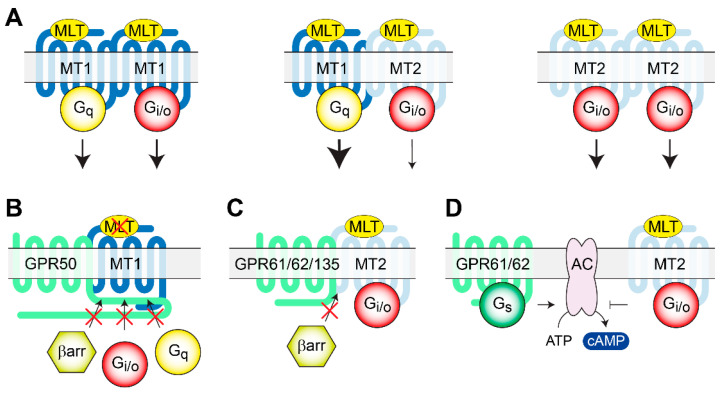
Heteromerization of melatonin receptors and oGPCRs. (**A**) MLT activation of homo- and heteromers of MT1 and MT2 receptors triggering Gi/o and Gq signaling pathways. (**B**) Heteromerization of MT1 with GPR50 inhibits melatonin binding to MT1 and recruitment of signaling molecules. (**C**) Similarly, MT2 heteromerization with GPR61, GPR62, or GPR135 inhibits β-arrestin recruitment but does not affect MLT binding, nor G protein activation. (**D**) The constitutive Gs signaling of GPR61 and GPR62 counteracts MT2 receptor Gi//o signaling, modulating intracellular cAMP levels. AC, Adenylate Cyclase; βarr, β arrestin; MLT, melatonin; MT1, melatonin receptor 1; MT2, melatonin receptor 2.

The location of the GPR50 gene on Xq28 [159], a chromosomal region with a link to BPD that is still debated, prompted human association studies between BPD, MDD, and schizophrenia with GPR50 [76,168]. An insertion/deletion polymorphism was located in GPR50 and designated GPR50^Δ502–505^; therefore, a case–control association study from a sampled population in Scotland was conducted [168]. The results of this study highlighted a significant association between this polymorphism and both MDD and BPD, but not with schizophrenia subjects [168]. Additionally, when subdivided into gender groups, BPD and MDD female subjects showed a very strong association, while there was no association with males, suggesting this association to be sex-specific [168]. A further association study found no linkage with parent of origin and early onset for female MDD subjects; however, female subjects with BPD and GPR50^Δ502–505^ showed an association with early age of onset but not with parent of origin [168]. A weak association with MDD female patients was also found for another nonsynonymous polymorphism, Val^606^Ile (rs13440581), but no association was observed in the male subjects [168]. Several other groups attempted this association study on various populations—Northern Swedish, Hungarian, French, and Scottish—along with different age groups, ranging from children to the elderly, with controversial outcomes [76,77,78,79,80]. Glucocorticoids (GCs) are a class of steroid hormones released by the adrenal cortex after the stress-induced activation of the HPA axis; once the GCs reach the brain, they activate the mineralocorticoid receptors, expressed in limbic regions, and glucocorticoid receptors, ubiquitously expressed across the brain [169]. Because of the large number of molecular pathways regulated by GCs, such as the rapid activation of CREB, promotion of epigenetic mechanisms, and the modulation of GABAergic/glutamatergic signaling [169], they have been widely exploited pharmacologically. Recently, a role of GPR50 has been linked to the glucocorticoid receptor (GCR) signaling pathways [170]. By utilizing approaches, including the yeast-two-hybrid screen, co-immunoprecipitation, and co-localization in HEK293 cells, Li and colleagues discovered that GPR50 acts as a modulator on Tat-interactive protein-60 kDa (TIP60). TIP60 is a transcriptional co-activator with histone acetyltransferase activity, which promotes the activity of a variety of transcription factors, including nuclear hormone receptors [171,172]. When coexpressed in HEK293 cells, both full-length GPR50 and TIP60 resulted in perinuclear localization; in addition, GPR50 was also still observed at the plasma membrane [170]. However, no change in localization was observed when TIP60 was coexpressed with a truncated version of GPR50; while, co-transfection with the C-terminal tail of GPR50 induced a translocation of both TIP60 and the C-terminal tail to the nuclear compartment [170]. GPR50 enhances the TIP60 coactivation of GCR signaling, thereby revealing a potential role of GPR50 in the GC modulation of transcriptional programs [170] implicated in a number of physiological processes [173]. GPR50 and GPR61 mouse model studies have mainly focused on their role in metabolism [174,175]; however, the mRNA levels of brain derived neurotrophic factor (BDNF) in the hypothalamus were found lower in the GPR61 deficient mice compared to the wild-type littermates [174]. This holds promise, since BDNF has been shown to contribute toward mood disorders [176], as shown in Figure 2. Further analysis using mouse models for this group of oGPCRs could provide pharmacological insights into the role of the melatonergic system in mood disorders. Finally, drug discovery research has recently developed bivalent ligands that specifically target heteromers [177]. The main advantage of this approach consists in the potentially limited side effects due to the high specificity of the target, and therefore represents a valuable method to exploit this family of GPCRs as a target for antidepressant therapy.

### 2.7. GPR52

GPR52 gene was identified in 1999 and found to have high homology with GPR21, histamine H2 receptor, and 5-HT4 receptor [178]. Twenty-one years later, the high-resolution crystal structure of human GPR52 was obtained in a ligand-free state, a Gs-coupled self-activation state, and a potential allosteric ligand-bound state [179]. Through the screening of a library of 5000 compounds using a mutated cyclic nucleotide-gated channel (CNG)-based assay [180], it was found that reserpine can activate this oGPCR [81]. This screening also provided the first evidence of GPR52 being coupled to Gαs [81]. Validation experiments using the same method in GPR52 transfected HEK293 cells showed that reserpine selectively and dose-dependently activated GPR52 [81]. Such coupling was later confirmed in transfected CHO cells expressing GPR52, where reserpine increased intracellular cAMP levels and allowed GFP-fused GPR52 to be internalized [81]. Preliminary expression studies on GPR52 showed enrichment in the brain, specifically in the striatum in humans, mice and rats; however, a detailed analysis by qRT-PCR in rat brains also established GPR52 expression in the medial PFC, basolateral amygdala, and habenula, all regions that are relevant in controlling emotional behaviors [81]. More specifically, GPR52 was found to co-express in the medial PFC with the dopamine D1 receptor and in the basal ganglia with the dopamine D2 receptor [81]. Because of this segregated expression pattern, it was hypothesized that the GPR52 activation of Gαs could stimulate cAMP production, counteracting the effect of Gαi-activation by dopamine D2 receptors [81]. Such a hypothesis led to the development of many synthetic ligands to be potentially used in the treatment of schizophrenia [181,182,183].

Since GPR52 expression is enriched in the brain, KO mouse models were established in order to elucidate its function in vivo [81]. OFT on a line of GPR52 KO mice showed a longer time spent in the center, suggesting a role of GPR52 in anxiety-like behaviors [81]. However, the observed hyperactivity of the GPR52 KO mice could have played a role in such findings; therefore, further and more directed analysis of anxiety-like behaviors should be conducted. Moreover, the hypothesized role of GPR52 in modulating the dopaminergic system indicates a possible involvement in the aggravating symptoms of mood disorders, such as anhedonia [4,184]. Another study revealed a modulation of GPR52 levels by stress hormones [82]. The stress-induced release of GCs from the adrenal cortex activates transcriptional programs aimed at modulating brain function [169]. Because of their profound effects, GCs have been used for pharmacotherapies; however, GC treatments lead to a multitude of changes: (1) an increased risk of developing stress-related behavioral problems (e.g., anxiety, MDD, hyperactivity, and bipolar personality disorder); (2) exposure to high levels of GCs results in gene expression changes that persist through adulthood (i.e., elements of the GABAergic signaling pathway) [185]. GCs are administered to women at risk of preterm delivery to facilitate fetal lung maturation [186]. Transcriptional research after prenatal synthetic GC exposure to pregnant guinea pigs found 10 genes being differentially expressed in the PFC of the progeny, both male and female in the first generation, and female in the second and third generation. These genes were ranked utilizing recursive feature selection based on their contribution to the observed variation in the OFT activity, with GPR52 expression being decreased in the GC-exposed offspring and ranked third [82]. Due to the altered expression levels, GPR52 seems to be involved in stress-related and anxiety processes, and further research could provide a more concrete understanding of this oGPCR’s neurophysiological role.

### 2.8. GPR88

GPR88 is one of the highest expressed GPCRs in the striatal medium spiny neurons [187], but it is also expressed at lower levels in other brain regions, including the PFC, CeA, thalamus, inferior olive, and bed nucleus of the stria terminalis [188,189,190,191,192]. Despite extensive efforts towards the deorphanization of GPR88, the endogenous ligand(s) remains unknown [193,194,195,196]. Nonetheless, the first synthetic agonists for this oGPCR have been identified, while high affinity antagonists are still unavailable [193,195,197]. Although GPR88 signaling properties still lack a detailed analysis, it was shown that the pharmacological stimulation of GPR88 led to the activation of G protein heterotrimers of the Gi/o/z family, both in native and heterologous expression systems [88,196,197]. The striatal enrichment of GPR88 inspired a number of studies that linked it to neurophysiological processes, such as motor control, reward behaviors, cognitive functions, and neuropsychiatric disorders resulting from deficits in striatal function [88,89,191,198,199,200].

**Figure 3:**
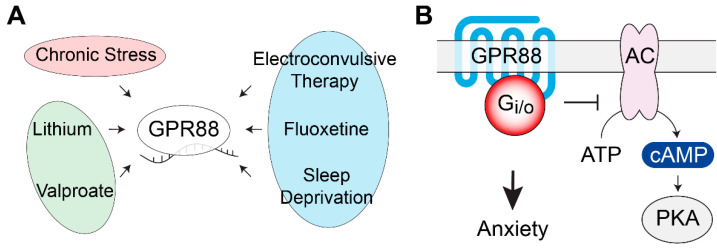
GPR88 gene expression regulation and signaling pathways. (**A**) GPR88 mRNA levels are modulated by antidepressant treatments, mood stabilizers, and by chronic stress in several brain regions. (**B**) Behavioral studies using conditional KO mice revealed the involvement of GPR88 expressed in the dopamine D2 receptor-expressing medium spiny neurons of the striatum in the development of anxiety-related phenotypes. AC, adenylate cyclase; PKA, protein kinase A.

The human GPR88 gene localization at 1p22-p21, a chromosomal region linked to BPD [201], prompted a GWAS that revealed a genetic association between BPD and GPR88 in the Sardinian and Palestinian populations [64]. Additionally, transcriptomic studies in rodents have shown that GPR88 expression is significantly up-regulated in the cortex and PFC by mood stabilizers, such as lithium or valproate, used for the treatment of BPD [85,86]. Similarly, Conti and colleagues discovered that two fast-onset antidepressant therapies, electro convulsive therapy and sleep deprivation, significantly increased GPR88 mRNA, while the slow-onset antidepressant fluoxetine reduced GPR88 expression levels in rat hypothalamus. Intriguingly, changes in both directions were also observed for only some of these treatments in specific brain regions: amygdala, hippocampus, locus coeruleus, and PFC [87]. On the other hand, it was shown that chronic physical restraint stress (PRS) in mice up-regulated GPR88 mRNA expression in the hippocampus, as measured by microarray and qRT-PCR [83]. Furthermore, a transcriptomic analysis validated by qRT-PCR in a mouse model of endometriosis-induced anxiety and depression identified GPR88 as the most significantly up-regulated gene in the insular cortex [84]. A further immunohistochemical analysis using this model showed a significantly higher percentage of GPR88 positive cells in the insular cortex. Among the behavioral features of these mice, there was a reduced time in the center of the arena in the OFT, and increased immobility in the TST [84]. Altogether, these findings suggest that GPR88 levels are finely regulated by sophisticated mechanisms that depend on the brain area and most likely involve a variety of signaling cascades. Considering the physiological relevance of GPR88, several lines of GPR88 KO mice were created to assess the gene function globally or in specific neuronal populations [88,89,90,202]. GPR88 has been shown to be densely expressed in the CeA, a brain region that controls emotional processing. This expression pattern prompted an analysis of anxiety-like related behaviors in global GPR88 KO mice that revealed markedly reduced levels of anxiety [88,89,90]. In detail, EPM revealed a longer time spent in the open arms for GPR88 KO mice; similarly, behavioral defensive responses that are sensitive to anxiolytic treatments, such as flat back approaches, stretched attend postures, and head dips, were more frequent in the GPR88 KO mice, suggesting altogether reduced anxiety levels [88]. This conclusion was further supported by a lower number of marbles buried in MBT. Moreover, using the NSFT to assesses the latency to eat familiar food in a novel environment, the GPR88 KO mice exhibited shorter latency to start eating in the center of the arena compared with the wild-type animals [88]. Interestingly, the chronic blockade of delta opioid receptors with naltrindole normalized the levels of anxiety in the GPR88 KO mice, indicating the involvement of opioid signaling [88]. Such behavioral observations prompted a neurochemistry analysis of the DA levels within the striatum that unveiled lower [198] or unchanged levels [191], probably due to a specific deficit in the dorsal striatum [88]. DA analysis across brain regions also revealed lower levels in the CeA but no differences in the PFC and hippocampus [88]. Finally, higher levels of 5-HT and NE were found at their respective sites of synthesis [88]. Further observations that may help our understanding of the behavioral effects of GPR88 ablations consisted in an altered neuronal morphology with a reduction in spine density in the striatum, amigdala, and CA1 regions of the hippocampus [88]. Lastly, it was shown that the inactivation of GPR88 increased medium spiny neuron excitability in the striatum by modulating RGS4-dependent GABAergic and glutamatergic signaling [191]. In an effort to dissect the neuronal contribution to the anxiety-related behavior observed in GPR88 KO mice, Meirsman and colleagues generated conditional KO animals to specifically ablate GPR88 in Dopamine D2 receptor- (A_2A_R-GPR88 KO) or dopamine D1 receptor-expressing (D1-GPR88 KO) medium spiny neurons of the striatum. They showed that the A_2A_R-GPR88 KO mice replicated the anxiolytic-like phenotype of the global GPR88 KO mice in LDT, MBT, and EPM, while they did not show a greater motivation to explore novel environments in the NFST, as observed in global GPR88 KO [89]. On the other side, D1-GPR88 KO did not show anxiety-related behaviors in the MBT test [90]. Furthermore, in a rat model of Parkinson’s disease, the lentiviral shRNA knockdown of GPR88 in the dorsomedial striatum showed an antidepressant-like effect in the FST, while GPR88 knockdown in the dorsomedial or dorsolateral striatum had no impact on SPT [91]. Based on the multitude of evidence pointing at GPR88 as a major player in anxiety-related behaviors, further studies are highly desirable to unravel the details of GPR88’s involvement in mood disorders and to interrogate its function pharmacologically, as shown in Figure 3.

### 2.9. GPR158

The oGPCR GPR158 shows unique topological features as it lacks the Venus flytrap ligand-binding domain typical of a class C GPCR, while it bears remarkably long extracellular N-terminus and intracellular C-terminus [203]. GPR158 has been proposed as the brain receptor for the bone-derived hormone, osteocalcin [93]. Immunoprecipitation studies indicated a physical interaction and suggested coupling with Gq [93]. Reduced IP1 accumulation in response to osteocalcin treatments of GPR158 KO hippocampal neuronal cultures, as well as altered the electrophysiological effects elicited by osteocalcin treatments in CA3 hippocampal neurons, supported the pairing of GPR158–osteocalcin [93]. However, a number of cellular and molecular adaptations that may be indirectly responsible for the observed altered osteocalcin responses have been reported in the hippocampus and PFC of GPR158 KO mice [92,94,204,205]. Functional in vitro studies are therefore necessary to confirm this ligand–receptor pairing. GPR158 ectodomain has also been shown to interact with several extracellular matrix components of the heparan sulfate proteoglycan family [204,206]. The functional consequences of these interactions on GPR158 signaling are yet to be explored. Proteomics studies identified a G protein-independent signaling mechanism for GPR158, mediated by its interaction with the obligatory heterodimer, Regulator of G protein Signaling 7 (RGS7)/Gβ5 [203], a strong negative modulator of Gi/o signaling. Specifically, GPR158 has been shown to control proteolytic stability, membrane targeting, and the catalytic activity of RGS7 [207], therefore, forming a unique membrane macromolecular complex that regulates signaling pathways initiated by many other GPCRs in vivo [95]. Finally, quantitative proteomics showed that GPR158 is particularly enriched in the PFC, where it is by far the most expressed oGPCR [92]. In the rest of the brain, GPR158 is also highly expressed in the striatum, amygdala, cerebellum, and hippocampus [93,94,203,204,208].

**Figure 4:**
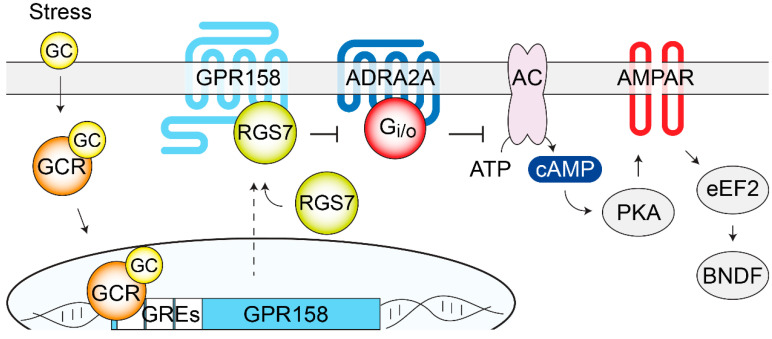
GPR158/RGS7 complex is a stress-induced modulator of depressive behaviors. GC released by stress bind to intracellular GCR in the mPFC and trigger GPR158 transcription. Increased amounts of GPR158 at the plasma membrane recruit additional RGS7 that regulates many canonical GPCRs (ADRA2A and GABABR have been described). Downstream signaling cascades, modulated by the GPR158/RGS7 complex, include cAMP-PKA, phosphorylation and expression levels of glutamate AMPA receptors, phosphorylation of eEF2, and BDNF local synthesis. Overall, these cellular adaptations are likely responsible for mood regulation. AC, adenylate cyclase; ADRA2A; α2-adrenergic receptor; AMPAR, α-amino-3-hydroxy-5-methyl-4-isoxazolepropionic acid; BDNF, brain derived neurotrophic factor; eEF2, eukaryotic elongation factor 2; GC, glucocorticoids; GCR, glucocorticoid receptor; GRE, glucocorticoid response elements; PKA, protein kinase A; RGS7, regulator of G protein signaling 7.

GPR158 has been recently identified as a key mediator of stress-induced depression, both in mouse models and humans [92,95,205]. A Western blot analysis of post-mortem dorsolateral PFC from diagnosed MDD patients demonstrated significantly higher levels of GPR158 compared to matched controls [92]. Subsequent mouse studies, fostered by the presence of three glucocorticoid response elements in the GPR158 promoter [209], revealed an up-regulation in GPR158 levels by corticosteroid treatments in vivo and in vitro [92]. Similarly, two different protocols of chronic stress, UCMS and PRS, resulted in GPR158 up-regulation in mouse medial PFC (mPFC); an effect that was abolished by the administration of the glucocorticoid receptor blocker RU486 during the stress protocol [92]. Remarkably, the viral overexpression of GPR158 in the mPFC was sufficient to induce a depressive-like state in mice, indicating a primary role in mood regulation played by GPR158, expressed specifically in the mPFC but not in other brain regions [92]. On the contrary, the global ablation of GPR158 expression in mice induced an antidepressant-like behavior [92]. Both male and female adult GPR158 KO mice have been tested in TST and FST revealing a significantly shorter immobility time, which indicates an antidepressant-like phenotype [92]. Notably, such behavior was rescued by the viral overexpression of GPR158 in the mPFC [92]. Furthermore, GPR158 KO mice demonstrated resiliency to chronic stress-induced depression in several behavioral paradigms that also included SPT for anhedonia [92]. Anxiety-like behaviors have also been explored in both male and female GPR158 KO mice using EPM and MBT, revealing an anxiolytic phenotype [92]. Interestingly, the viral up-regulation of GPR158 in the mPFC did not rescue anxiolytic behaviors, indicating that GPR158 expressed in other brain regions must be involved [92]. Opposite results were, however, reported by another group investigating female GPR158 KO mice with EPM, LDT, and OFT [93], while a third research group did not detect any significant difference between the GPR158 KO and wild-type littermates using the OFT [94]. Further research aimed at clarifying the role of GPR158 in anxious behaviors is therefore required. At the cellular level, stress specifically up-regulated GPR158 in the glutamatergic neurons of the mPFC, while the GPR158 levels in GABAergic neurons were not affected [92]. Interestingly, no differences were observed in monoamine levels, or turnover, in the mPFC of the GPR158 KO mice [92]. At the molecular level, it has been demonstrated that GPR158 and RGS7 act as a unique complex in controlling behavioral adaptations, which lead to the modulation of emotional states [92,95]. In fact, male and female RGS7 KO mice showed a phenotype that was identical to what was described for GPR158 KO mice such as antidepressant-like and anxiolytic-like behaviors and resiliency to chronic stress [95]. Notably, the viral down-regulation of RGS7 in the mPFC produced an antidepressant-like phenotype, while RGS7 viral overexpression induced a depressive-like state, as measured by TST and FST [95]. To explore the role played by GPR158 in mediating the behavioral effects observed in RGS7 KO mice, the viral overexpression of RGS7 in the mPFC of GPR158 KO mice was performed, and it was not sufficient to reverse the antidepressant-like phenotype [95]. Moreover, the chronic stress-induced up-regulation of GPR158 in the mPFC augmented the membrane targeting of RGS7 only in the wild-type and not in the GPR158 KO animals [95]. These results point at RGS7 as a key mediator of GPR158 action in controlling depressive-related behaviors. As RGS7 is a known regulator of many GPCRs that have been involved in the action of a variety of antidepressants, pharmacological studies have been performed showing how the inhibition of the GABAB receptors or ADRA2A receptors was sufficient to reverse the antidepressant-like phenotype in GPR158 KO mice [95]. Further evidence from both the GPR158 KO and RGS7 KO studies implicated the intracellular elevation of cAMP levels and changes in the AMPA receptor subunit composition and phosphorylation state, which led to increased AMPA receptor currents [92,95]. The biochemical analysis of signaling proteins involved in the action of antidepressants revealed significant changes in the phosphorylation state of GSK3 and CaMKII in GPR158 KO mice [206]. Moreover, higher BDNF protein levels, that were not paralleled by changes in mRNA levels, were observed in GPR158 KO mPFC, suggesting a regulation of local BDNF translation, a hypothesis supported by a lower phosphorylation of the eukaryotic elongation factor 2 (eEF2) [92]. BDNF was likely responsible for the greater spine density assessed in the mPFC and hippocampal neurons of the GPR158 KO mice [92,204]. Notably, the reported changes in the levels and phosphorylation states of the signaling proteins, together with the alterations in neuronal morphology, are hallmarks of the action of antidepressant treatments and therefore can contribute to the elucidation of the antidepressant-like behaviors of the GPR158 KO mice [210,211], as shown in Figure 4. Overall, the atypical GPR158–RGS7 complex seems to act as a regulator of traditional GPCRs, which are involved in mediating antidepressant effects, by controlling their volume of G protein signaling. Developing pharmacological means aimed at breaking the complex formation between GPR158 and RGS7 holds promise for novel antidepressant therapies.

### 2.10. GPRC5B

The four evolutionary conserved oGPCRs, GPRC5(A–D), were originally described as Retinoic Acid-Inducible GPCRs, or RAIGs [212,213,214]. They are phylogenetically classified as class C GPCRs, however, they bear short N terminal domains lacking the ligand-binding Venus flytrap domain [215]. Each member exhibits unique tissue distribution and association to distinct pathological events, ranging from several cancer types to diabetes, atherosclerosis, inflammation, renal dysfunction, and neuropsychiatric conditions [216,217,218,219,220]. Despite their clear pathophysiological relevance, GPRC5(A–D) activation mechanisms and cellular signaling properties have yet to be characterized. GPRC5B is ubiquitously expressed, but is enriched in the brain where it controls motor learning and spontaneous activity in response to novel environmental cues, according to behavioral studies using a line of global GPRC5B KO mice [218,219]. Quantitative RT-PCR analysis across human tissues and rat brain regions, as well as the X-gal staining of sagittal mouse brain sections from GPRC5B heterozygous knockin mice, revealed an enrichment of GPRC5B in the neuronal cells of the hippocampus, amygdala, PFC, cerebellum, striatum, olfactory bulb, substantia nigra, thalamus, and the glial cells of the fiber tracts [215,218,221]. GPRC5B coupling to G12/13 was supported by in vitro competition studies, overexpressing C terminal peptides derived from four Gα-subunits (Gαs-CT, Gαq-CT, Gα12-CT, and Gα13-CT), or the Gαi/o inhibitor PTX. In such experiments, overexpressing GPRC5B induced the rounding of COS-7 cells that were prevented by coexpression with only Gα12-CT and Gα13-CT [222].

A post-mortem microarray gene expression analysis of the temporal cortex from MDD patients revealed a lower expression of GPRC5B [69]. A similar transcriptional analysis of the dorsolateral PFC and ACC, obtained post-mortem from patients with a diagnosis of either MDD or BPD, revealed changes in the levels of many G protein signaling genes [59]. Among these genes, GPRC5B expression was found to be significantly lower in MDD, and significantly higher in BPD compared to the controls in both areas analyzed [59]. These results were later confirmed by qRT-PCR in the ACC, showing a comparable alteration in gene expression [59]. This oGPCR is highly expressed in the central nervous system and represents an intriguing candidate for further animal research, aimed at defining its role in stress-related phenotypes.

## 3. Concluding Remarks

Currently available pharmacological antidepressant treatments are limited by their lack of effectiveness in half of the patients and by the delay in clinical response that may require weeks to months to become effective. Other shortcomings include severe side effects that, in many cases, affect long-term compliance with the treatment [223,224]. Some of these issues have been recently addressed by the development of a new class of fast-acting antidepressants targeting the glutamatergic system. In this direction, the US food and drug administration recently approved the use of ketamine for patients with intractable depression [19,20]. However, ketamine use as an antidepressant also has severe limitations due to the hallucinogenic and anesthetic effects that can be achieved at higher doses, and the risk of fatal overdoses, thereby restricting its use to a clinical setting [225,226]. Similarly, mood stabilizers for the treatment of BPD are associated with severe side effects, which highlights the need to develop more effective, tolerable, and safe treatments [227,228]. Altogether, basic research, aimed at understanding the molecular mechanisms leading to deficits in neurotransmitter/hormone systems, brain connectivity, and morphological alterations observed in affective disorders, is pivotal to improve the available therapies. In this context, oGPCRs should represent a primary research interest as they are involved in the neurophysiological processes regulating the perception of emotions, as they regulate or are regulated by stress responses, and, overall, as they mediate some of the neurotransmitter-activated signaling pathways that control mood and anxiety.

In addition to the oGPCRs discussed in this review, a few more have been proposed to be activated by endogenous ligands that were previously implicated in anxiety and mood disorders. Among these oGPCRs, GPR160 has recently been suggested as the receptor for cocaine- and amphetamine-regulated transcript peptide (CARTp), a widely distributed ligand in the central nervous system [229]. At the molecular level, CARTp was previously shown to activate an undefined Gi/o-coupled receptor [230], while the treatments of cells expressing endogenous GPR160 stimulated ERK phosphorylation, an effect that was attenuated by co-transfection with GPR160-specific siRNA [229]. Although the direct role of GPR160 in controlling depressive-like behavior has not been explored, there is evidence pointing at CARTp in the regulation of stress responses and anxiety [231,232,233,234]. Pending confirmation of the activation of GPR160 by CARTp, research into the involvement of GPR160 in anxiety and mood-related behaviors could possibly uncover a relevant pharmacological target. Similarly, two independent groups reported the association of GPR173 with the neuropeptide phoenixin [235,236]; however, evidence of direct interaction and G protein coupling are still absent and are currently being pursued [237]. Both animal and human studies have described the role of phoenixin in the modulation of anxiety. Specifically, it has been suggested that phoenixin mediates this effect through GPR173 expressed in hypothalamic GnRH neurons [238]. Again, a detailed investigation of the contribution of GPR173 to the pathophysiology of anxiety has not been reported yet.

The characterization of the cellular and molecular processes that control emotional behaviors is a fundamental challenge to developing effective therapeutics. The latest investigations into the rapid-acting and sustained effects of ketamine have helped discover new pathways that can lead to the identification of novel pharmacological targets [22,239]. However, a comprehensive theory of the neurobiology of mood disorders is still unavailable. Overall, studying the neurophysiological role of oGPCRs represents a vital challenge aimed at understanding the biological basis of neuropsychiatric disorders, while revealing.

## Figures and Tables

**Table 1:** Results of human and animal studies indicating the involvement of oGPCRs in mood and anxiety disorders.

oGPCR	Alias	Human Studies	Animal Studies
ADGRB2	BAI2		OFT, male ADGRB2 KO vs. WT mice, 11–14 week-old, ↔ time in center [65]
			TST, male ADGRB2 KO vs. WT mice, 11–14 week-old, ↓ immobility [65]
			SDT, male ADGRB2 KO vs. WT mice, 11–14 week-old, ↑ motor activity [65]
ADGRG1	GPR56	MDD antidepressant responders vs. non-responders, blood, microarray, ↑ mRNA levels [66].	Male mice, 3–6 month-old, blood, microarray, ↓ mRNA by UCMS rescued by treatments only in responders [66].
		MDD vs. controls, dlPFC, microarray, ↓ mRNA levels [59].)	Male mice, 3–6 month-old, PFC, microarray, ↓ mRNA by UCMS rescued by treatments only in responders [66].
		MDD vs. controls, PFC, RT-PCR, ↓ mRNA levels [66].	TST, male ADGRG1 OE in mPFC vs. control mice, 3–6 month-old, ↓ immobility [66].
			TST, male ADGRG1 KD in mPFC vs. control mice, 3–6 month-old, ↑ immobility [66].
			FST, male ADGRG1 OE in mPFC vs. control mice, 3–6 month-old, ↓ immobility [66].)
			FST, male ADGRG1 KD in mPFC vs. control mice, 3–6 month-old, ↑ immobility [66].)
			SPT, male ADGRG1 OE in mPFC vs. control mice, 3–6 month-old, ↔ ratio [66].
			SPT, male ADGRG1 KD in mPFC vs. control mice, 3–6 month-old, ↓ ratio [66].
			EPM, male ADGRG1 OE in mPFC vs. control mice, 3–6 month-old, ↔ immobility [66].
			EPM, male ADGRG1 KD in mPFC vs. control mice, 3–6 month-old, ↓ immobility [66].)
			TST, male ADGRG1 KD in mPFC vs. control mice, 3–6 month-old, ↔ immobility after fluoxetine [66].)
			TST, male, agonist infusion in mPFC, 3–6 month-old, ↓ immobility [66].
GPR3	ACCA		OFT, male GPR3 KO vs. WT mice, 2–6 month-old, ↓ exploratory behavior and time in the center [67].
			EPM, male GPR3 KO vs. WT mice, 2–6 month-old, ↓ time in open arms [67].
			FST, male GPR3 KO vs. WT mice, 2–6 month-old, ↑ immobility [67].)
			TST, male GPR3 KO vs. WT mice, 2–6 month-old, ↑ immobility [67].
GPR26			OFT, male GPR26 KO vs. Het vs. WT mice, 10–12 week-old, ↓ time in center - Het and KO [68]
			EPM, male GPR26 KO vs. Het vs. WT mice, 10–12 week-old, ↓ time in open arms - KO [68]
			FST, male GPR26 KO vs. Het vs. WT mice, 10–12 week-old, ↔ immobility - KO (P = 0.051) [68]
			TST, male GPR26 KO vs. Het vs. WT mice, 10–12 week-old, ↑ immobility - KO [68]
			EPP, male GPR26 KO vs. WT mice, 10–12 week-old, ↑ preference - 7% solution [68]
GPR37	PAELR	MDD vs. controls, frontotemporal cortex, microarray, ↓ levels [69]	Male mice, 8 week-old, amygdala, microarray, ↓ mRNA by UCMS rescued by treatments [70].
		MDD vs. controls, amygdala, microarray, ↓ levels [71].	EPM, female GPR37 KO vs. WT mice mice, 4–6 month-old, ↔ time in open arms [72].
		MDD vs. controls, dlPFC, microarray, ↓ levels [59].	EPM, male GPR37 KO vs. WT mice, 16–18 month-old, ↔ time in open arms [72].
		MDD vs. controls, ACC, microarray, ↓ levels [59].	EPM, female GPR37 KO vs. WT mice, 16–18 month-old, ↓ time in open arms [72].
		MDD vs. controls, ACC, RT-PCR, ↓ levels [59].	FST, female GPR37 KO vs. WT mice, 4–6 month-old, ↔ immobility [72].
		BPD vs. controls, dlPFC, microarray, ↑ levels [59].	FST, male GPR37 KO vs. WT mice, 16–18 month-old, ↔ immobility [72].
		BPD vs. controls, ACC, microarray, ↑ levels [59].	FST, female GPR37 KO vs. WT mice, 16–18 month-old, ↑ immobility [72].
			OFT, female GPR37 KO vs. WT mice, 16–18 month-old, ↔ time in center [72].
			LDT, female GPR37 KO vs. WT mice, 16–18 month-old, ↔ transitions [72].
			EPM, male GPR37 KO vs. WT mice, 8 week-old, ↑ time in open arms [73].
			MBT, male GPR37 KO vs. WT mice, 8 week-old, ↓ marble buried [73].
			OFT, male, 3 month-old mice, ChR2-GPR37 activation in dorsomedial striatum, ↑ time in center [74].
GPR50	H9, ML1X	Genetic linkage analysis, association between GPR50 and BPD & MDD [75].	
		Genetic linkage analysis, no association between GPR50 and BPD [76].	
		Genetic linkage analysis, no association between GPR50 and BPD & MDD [77].	
		Genetic linkage analysis, association between GPR50 and BPD & MDD [78].	
		Genetic linkage analysis, association between GPR50 and SAD [79].	
		Genetic linkage analysis, weak association between GPR50 and late-life depression [80].	
GPR52			OFT, GPR52 KO vs. WT mice, ↑ time in center [81].
			Transcriptomic analysis, guinea pigs, association between sGC administration and GPR52 expression [82].
GPR88		GWAS, genetic link between GPR88 and BPD [64].	Male mice, 2 month-old, hippocampus, microarray and RT-PCR, ↑ mRNA by chronic PRS [83].
			Mouse model of endometriosis-induced anxiety and depression, female, 9 week-old, amygdala, microarray and RT-PCR, ↑ mRNA [84].
			Male rats, 7–8 week-old, cortex, microarray, ↑ mRNA by lithium [85].
			Male mice, 8–12 week-old, PFC, microarray, ↑ mRNA by valproate [86].
			Male rats, adult, hypothalamus, microarray, ↑ mRNA by sleep deprivation [87].
			Male rats, adult, hypothalamus, microarray, ↑ mRNA by electro convulsive therapy [87].
			Male rats, adult, hypothalamus, microarray, ↓ mRNA by fluoxetine [87].
			EPM, male GPR88 KO vs. WT mice, 8–15 week-old, ↑ time in open arms [88],[89], [90]
			MBT, male GPR88 KO vs. WT mice, 8–15 week-old, ↓ marble buried [88], [89], [90]
			NSFT, male GPR88 KO vs. WT mice, 8–10 week-old, ↓ latency to feed [88]
			LDT, male GPR88 KO vs. WT mice, 9–15 week-old, ↑ time in lit box [89]
			LDT, male A2AR-GPR88 KO vs. GPR88flx/flx mice, 9–15 week-old, ↑ time in lit box [89]
			EPM, male A2AR-GPR88 KO vs. GPR88flx/flx mice, 9–15 week-old, ↑ time in open arms [89]
			MBT, male A2AR-GPR88 KO vs. GPR88flx/flx mice, 9–15 week-old, ↓ marble buried [89], [90]
			NFST, male A2AR-GPR88 KO vs. GPR88flx/flx mice, 9–15 week-old, ↔ latency to feed [89]
			MBT, male D1R-GPR88 KO vs. GPR88flx/flx mice, 9–15 week-old, ↔ marble buried [90]
			FST, male GPR88 KD in DMS vs. CNT rats, 7 week-old, ↓ immobility [91]
			SPT, male GPR88 KD in DMS vs. CNT rats, 7 week-old, ↔ ratio [91]
GPR158		MDD vs. controls, dlPFC, WB, ↑ levels [92]	Male mice, 2–4 month-old, mPFC, WB, ↑ levels by chronic PRS, blocked by RU-486 [92]
			Male mice, 2–4 month-old, mPFC, WB, ↑ levels by UCMS [92]
			Male mice, 2–4 month-old, mPFC, WB, ↑ levels by chronic corticosterone [92]
			Primary cortical neurons, WB, ↑ levels by chronic corticosterone [92]
			TST, male GPR158 KO vs. WT mice, 2–4 month-old, ↓ immobility, rescued by viral GPR158 OE in mPFC [92]
			TST, female GPR158 KO vs. WT mice, 2–4 month-old, ↓ immobility [92]
			TST, male GPR158 OE in mPFC vs. control mice, 2–4 month-old, ↑ immobility [92]
			FST, male GPR158 KO vs. WT mice, 2–4 month-old, ↓ immobility, rescued by viral GPR158 OE in mPFC [92]
			FST, female GPR158 KO vs. WT mice, 2–4 month-old, ↓ immobility [92]
			FST, male GPR158 OE in mPFC vs. control mice, 2–4 month-old, ↑ immobility [92]
			EPM, male GPR158 KO vs. WT mice, 2–4 month-old, ↑ time in open arms [92]
			EPM, female GPR158 KO vs. WT mice, 2–4 month-old, ↑ time in open arms [92]
			EPM, female GPR158 KO vs. WT mice, 3 month-old, ↓ time in open arms [92]
			MBT, male GPR158 KO vs. WT mice, 2–4 month-old, ↓ marble buried [92]
			MBT, female GPR158 KO vs. WT mice, 2–4 month-old, ↓ marble buried [92]
			LDT, female GPR158 KO vs. WT mice, 3 month-old, ↓ time in lit box [93]
			OFT, female GPR158 KO vs. WT mice, 3 month-old, ↓ time in center [93]
			OFT, male GPR158 KO vs. WT mice, 8–12 week-old, ↔ time in center [94]
			TST after UCMS, GPR158 KO vs. WT mice, 2–4 month-old, ↔ immobility time [92]
			FST after UCMS, GPR158 KO vs. WT mice, 2–4 month-old, ↔ immobility time [92]
			EPM after UCMS, GPR158 KO vs. WT mice, 2–4 month-old, ↔ time in open arms [92]
			MBT after UCMS, GPR158 KO vs. WT mice, 2–4 month-old, ↔ marble buried [92]
			SPT after UCMS, GPR158 KO vs. WT mice, 2–4 month-old, ↔ sucrose preference [92]
			TST, male GPR158 KO vs. WT mice, 2–4 month-old, ↓ immobility after vehicle injection [95]
			TST, male GPR158 KO vs. WT mice, 2–4 month-old, ↔ immobility time after yohimbine injection [95]
			TST, male GPR158 KO vs. WT mice, 2–4 month-old, ↔ immobility time after CGP35348 injection [95]
			FST, male GPR158 KO vs. WT mice, 2–4 month-old, ↓ immobility after vehicle injection [95]
			FST, male GPR158 KO vs. WT mice, 2–4 month-old, ↔ immobility time after yohimbine injection [95])
			FST, male GPR158 KO vs. WT mice, 2–4 month-old, ↔ immobility time after CGP35348 injection [95]
GPRC5B	RAIG-2	MDD vs. controls, frontotemporal cortex, microarray, ↓ levels [69].	
		MDD vs. controls, dlPFC, microarray, ↓ levels [59].	
		MDD vs. controls, ACC, microarray, ↓ levels [59].	
		MDD vs. controls, ACC, RT-PCR, ↓ levels [59].	
		BPD vs. controls, dlPFC, microarray, ↑ levels [59].	
		BPD vs. controls, ACC, microarray, ↑ levels [59].	

MDD, major depressive disorder; BPD, bipolar disorder; SAD, seasonal affective disorder; dlPFC, dorsolateral prefrontal cortex; mPFC, medial prefrontal cortex; ACC, anterior cingulate cortex; DMS, dorsomedial striatum; WT, wild-type; KO, knockout; KD, knockdown; Het, heterozygus; OE, overexpression; UCMS; unpredictable chronic mild stress; PRS, physical restraint stress; TST, tail suspension test; FST forced swim test; EPM, elevated plus maze; OFT, open field test; SPT, sucrose preference test; LDT, light-dark transition; MBT, marble burying test; SDT, social defeat test; EPP, ethanol preference procedure; RT-PCR, real time polymerase chain reaction; WB, western blot.
